# Effects of Nutritional Mode on the Physiological and Biochemical Characteristics of the Mixotrophic Flagellate *Poterioochromonas malhamensis* and the Potential Ecological Implications

**DOI:** 10.3390/microorganisms10050852

**Published:** 2022-04-20

**Authors:** Mingyang Ma, Chaojun Wei, Man Chen, Hongxia Wang, Yingchun Gong, Qiang Hu

**Affiliations:** 1 Center for Microalgal Biotechnology and Biofuels, Institute of Hydrobiology, Chinese Academy of Sciences, Wuhan 430072, China; mamingyang@ihb.ac.cn (M.M.); chaojunwei@ihb.ac.cn (C.W.); manchen@ihb.ac.cn (M.C.); hongxiawang@ihb.ac.cn (H.W.); 2 Key Laboratory for Algal Biology, Institute of Hydrobiology, Chinese Academy of Sciences, Wuhan 430072, China; 3 Institute for Advanced Study, Shenzhen University, Shenzhen 518060, China; 4 Hydrobiological Data Analysis Center, Institute of Hydrobiology, Chinese Academy of Sciences, Wuhan 430072, China

**Keywords:** *Poterioochromonas malhamensis*, mixotrophic flagellate, biochemical, nutritional mode, feeding

## Abstract

Mixotrophic flagellates play an important role in connecting the classical food chain and microbial food loop. The feeding characteristics of the mixotrophic flagellate *Poterioochromonas* *malhamensis* have been well studied, but its role as a food source for other large zooplankton is less studied. This study focuses on the physiological and biochemical changes in *P. malhamensis* when using autotrophy, chemoheterotrophy, and phagotrophy, and the effect of these changes on the feeding ability of one of its predators, the ciliate *Paramecium caudatum*. The results showed that chemoheterotrophic *P. malhamensis* had a higher growth rate and larger cell size than autotrophic and phagotrophic *P. malhamensis*. The biochemical composition of *P. malhamensis* also varied greatly between the three nutritional modes. The protein, total absolute amino acid, and fucoxanthin contents were highest for autotrophic *P. malhamensis*, while chemoheterotrophic *P. malhamensis* had the highest contents of total sugar and total absolute fatty acid. The contents of most biochemical components in phagotrophic *P. malhamensis* fell between those in autotrophic and chemoheterotrophic *P. malhamensis*. A feeding experiment showed that the grazing ability of *P. caudatum* on chemoheterotrophic *P. malhamensis* was significantly higher than that on phagotrophic *P. malhamensis* and autotrophic *P. malhamensis*. This study showed that the transformation of nutritional modes can alter the biochemical composition of the mixotrophic flagellate *P. malhamensis* and, as a result, affect the grazing ability of its predator *P. caudatum*.

## 1. Introduction

Mixotrophy—the combination of autotrophy and heterotrophy, the latter meaning utilization of dissolved organic substrates (i.e., osmotrophy) or grazing on particulate organic matter (i.e., phagotrophy)—is a common nutritional mode in algal protists [1,2]. Among the different types of mixotrophs, some mixotrophic flagellates (mainly the members of the Chrysophyceae, Dinophyceae, Cryptophyceae, and Dictyochophyceae) are capable of living by photosynthesis, uptake of dissolved organic substrates, and grazing on particulate food simultaneously [3,4]. These mixotrophic flagellates are widely distributed in marine or freshwater ecosystems and play an important role in the cycling of matter and the flow of energy in natural ecosystems [5,6,7]. They are an important component of the primary producers, and in some acidic lakes they can dominate the plankton [8]. Furthermore, they can graze on a large number of bacteria or phototrophic pico-phytoplankton and then, in turn, be consumed as food by zooplankton or fish. These mixotrophic flagellates are, therefore, an important link connecting the classical food chain and the microbial loop [9]. However, most studies only focus on their importance as bacterivores or algivores [10,11], while their role as a food source for other large zooplankton is less studied.

The chrysophyte *Poterioochromonas*, which has two unequal flagella and one golden-brown plastid, has been widely studied as a model mixotrophic microorganism. *Poterioochromonas* has a wide distribution globally and has been found in many aquatic environments [12]. Through previous studies, the role of *Poterioochromonas* as a predator and its feeding characteristics are well known. For example, *Poterioochromonas malhamensis* has proved to be an omnivorous grazer that can graze on a range of bacteria and microalgae [13], with its feeding ability largely influenced by the size and shape of prey, environmental temperature, and pH value [12,14,15]. On the other hand, *P. malhamensis* can also be consumed by large zooplankton, such as *Daphnia magna* and the rotifer *Brachionus angularis* [16,17]. However, the factors affecting *P. malhamensis* as a food source for large zooplankton remain less studied.

The morphological characteristics and biochemical composition of prey are generally considered as the two main factors that influence the feeding behavior of large zooplankton [18,19]. It is well known that the biochemical composition of predators is closely correlated with the biochemical composition of their prey. In addition, the physiological and biochemical composition of mixotrophic microorganisms is largely dependent on the nutritional conditions in their environment. For instance, *Chlorella* cells can accumulate a large amount of lipid during the transition from heterotrophic to autotrophic nutrition [20], and autotrophic *Chlamydomonas* cells can have a lower content of protein than that found in heterotrophic cells [21]. Changes in the physiological characteristics and biochemical composition of mixotrophic microorganisms when they change to a different trophic mode are therefore expected to further influence the feeding behavior of their predators.

*Paramecium caudatum*, a free-living transparent unicellular microorganism, is widely distributed in freshwater ecosystems. It is omnivorous, and its diet consists of bacteria, microalgae, and yeast [22]. It is, however, also the high-quality prey of other zooplankton and fish larvae. Therefore, *P. caudatum* is an important model microorganism for research into the food-chains of aquatic ecosystems [23]. In this study, we hypothesize that the transformation of nutritional modes can alter the physiological and/or biochemical characteristic of the mixotrophic flagellate *P. malhamensis* and further affect the grazing ability of its predator *P. caudatum*. To test this hypothesis, we first establish the axenic culture of *P. malhamensis* isolated from *Chlorella* culture and then explore the effect of nutritional mode on the physiological and biochemical characteristics of *P. malhamensis*. Furthermore, the feeding abilities of *P. caudatum* on *P. malhamensis* in its different trophic modes are determined. Based on these results, we discuss the relationship between the feeding ability of *P. caudatum* on *P. malhamensis* and the physiological and biochemical characteristics of *P. malhamensis*.

## 2. Materials and Methods

### 2.1. Isolation and Cultivation of P. malhamensis

The mixotrophic flagellate *P. malhamensis* was isolated from a collapsed culture of *Chlorella sorokiniana* GT-1 [12] and was preserved in the China General Microbiological Culture Collection Center (CGMCC, No. 11620). The isolated flagellate was cultivated in autotrophic AF-6 medium [24] with a culture temperature of 22–25 °C and a light intensity of 30–50 μmol photons m^−2^ s^−1^ maintained on a 24:0 L:D cycle. The AF-6 medium consisted of: (1) NaNO_3_ (140 mg L^−1^), NH_4_NO_3_ (22 mg L^−1^), MgSO_4_·7H_2_O (30 mg L^−1^), KH_2_PO_4_ (10 mg L^−1^), K_2_HPO_4_ (5 mg L^−1^), CaCl_2_·2H_2_O (10 mg L^−1^), CaCO_3_ (10 mg L^−1^), citric acid (2 mg L^−1^), and Fe-citrate (2 mg L^−1^); (2) biotin (0.002 mg L^−1^), vitamin B_1_ (0.01 mg L^−1^), vitamin B_6_ (0.001 mg L^−1^), and vitamin B_12_ (0.001 mg L^−1^); and (3) Na_2_EDTA·2H_2_O (3.75 mg L^−1^), FeCl_3_·6H_2_O (0.485 mg L^−1^), MnCl_2_·4H_2_O (0.205 mg L^−1^), ZnCl_2_·7H_2_O (0.025 mg L^−1^), CoCl_2_·6H_2_O (0.01 mg L^−1^), and Na_2_MoO_4_·2H_2_O (0.02 mg L^−1^).

### 2.2. Purification of P. malhamensis Culture

The original culture of *P. malhamensis* was found to contain bacterial contaminants, and so the seed culture of *P. malhamensis* was purified using a modification of the method of [25,26], in which *P. malhamensis* culture contaminated by bacteria was repeatedly treated with mixed antibiotics, followed by the wide-spectrum antibiotic ciprofloxacin. The mixed antibiotics consisted of streptomycin, chloramphenicol, and gentamicin, with a weight ratio of 1:1:0.5. The final concentrations of the mixed antibiotics and the wide-spectrum antibiotic were all 100 mg L^−1^. To obtain a high cell concentration, the flagellate *P. malhamensis* was cultivated in BG-11 medium [27] containing killed cells of the prey bacterium *Bacillus sabtilis* at a concentration of 10^7^ cells mL^−1^. The *B. sabtilis* was isolated from a contaminated *Scenedesmus acuminatus* culture and preserved in our laboratory. The killed bacteria were prepared by subjecting live bacteria to a temperature of 121 °C for 15 min to achieve cell death.

The purification process began by transferring aliquots of the original *P. malhamensis* culture into 24-well plates with 1 mL per well. The *P. malhamensis* was first treated with the mixed antibiotics at 10–20 μmol photons m^−2^ s^−1^ for 12–14 h. The supernatant from the treated *P. malhamensis* cultures was then transferred into BG-11 medium containing the killed bacteria. After substantial proliferation, the high-concentration *P. malhamensis* cultures were then treated with the wide-spectrum antibiotic. The purification process using the wide-spectrum antibiotic was basically the same as that using mixed antibiotics except for the antibiotic used. The purified *P. malhamensis* cultures were examined under light microscopy (BX53, Olympus, Tokyo, Japan) to check for bacteria. The treatment of mixed antibiotics and a wide-spectrum antibiotic was repeated 2–3 times until no bacteria were observed, indicating that an axenic culture of *P. malhamensis* had been obtained. To confirm its axenic status, this culture was then cultivated with an enriched medium consisting of tryptone (1 g L^−1^), yeast extract (1 g L^−1^), beef liver extract powder (1 g L^−1^), and glucose (10 g L^−1^).

### 2.3. Cultivation of P. malhamensis with Different Nutritional Modes

Axenic *P. malhamensis* was cultivated under three sets of nutritional conditions, each promoting one mode of nutrition, i.e., autotrophy, chemoheterotrophy, and phagotrophy. For autotrophic growth, the culture medium was AF-6 medium. For chemoheterotrophic growth, *P. malhamensis* was cultivated in the enriched medium. For phagotrophic growth, *P. malhamensis* was cultivated in BG-11 medium containing prey *Chlorella sorokiniana* cells at a concentration of 1.6 × 10^7^ cells mL^−1^. According to microscopic observation, few *C. sorokiniana* cells were left after feeding by *P. malhamensis* for 10 days. It should be noted that the seeds for the different nutritional modes were all from chemoheterotrophic *P. malhamensis*. For each of the three nutritional modes, the culture system was 250 mL flasks, set up in quadruplicate. In each case, the initial cell concentration of *P. malhamensis* was 3 × 10^5^ cells mL^−1^. The temperature of the culture room was 21–23 °C. The cultures were all kept at an illumination of 40 μmol photons m^−2^ s^−1^, which was because *P. malhamensis* in continuous darkness cannot grow well for longer than 5 days and would die after 10 days [14,15]. The prevailing nutritional mode of the mixotrophic flagellate *P. malhamensis* in the coexistence of light and organic matter is heterotrophy [28,29]. Therefore, in this study, chemoheterotrophy and phagotrophy actually represented ‘predominantly chemoheterotrophic mixotrophy’ and ‘predominantly phagotrophic mixotrophy’, respectively.

### 2.4. Growth and Cell Morphology of P. malhamensis with Different Nutritional Modes

The cell concentration of *P. malhamensis* for each of the three nutritional modes was measured daily over a 10-day period using a hemocytometer (Improved Neubauer, Marienfeld, Thuringia, Germany) under light microscopy (CX31, Olympus, Tokyo, Japan) at 400× magnification. Lugol’s iodine with a final concentration of 1% [30] was used to stain the *P. malhamensis* cells before counting. Cell sizes of *P. malhamensis* were determined for each nutritional mode every other day using a light microscope equipped with a measurement function (BX53, Olympus, Tokyo, Japan). For each sample, at least 100 *P. malhamensis* cells were measured to calculate the mean value. In addition, the BX53 microscope was also used to observe and record the cell morphologies of *P. malhamensis* for each nutritional mode every other day.

### 2.5. Analysis of Biochemical Composition of P. malhamensis with Different Nutritional Modes

The biochemical composition of *P. malhamensis*, including total lipid, total protein, total sugar, fucoxanthin, amino acid, and fatty acid, was analyzed. The analysis was performed using *P. malhamensis* cells harvested by centrifugation after cultivation for 10 days. Prior to analysis, the harvested cells were freeze-dried using a lyophilizer (Labconco, Freezone^®^, Kansas City, MO, USA) to create a powder. Each sample was determined in triplicate.

The analytical methods for the total protein content and total sugar content were as previously described [31]. The total lipid content was measured according to [32] with a slight modification. The lipid was extracted from dried sample (50–100 mg) using chloroform:methanol (2:1), with violent shaking at room temperature for 1 h, and then separated into aqueous, powder residue and organic layers by the addition of 1.5 mL 0.7% KCl. The lowest organic layer was carefully transferred into a new vial after the extract was centrifuged at 1000× *g* for 10 min. The organic layer was then evaporated using nitrogen gas. The dried lipid was resuspended with 1 mL n-hexane and transferred into a new pre-weighed centrifuge tube. After drying with nitrogen gas, the increased weight of the centrifuge tube was the weight of extracted lipid. The ratio of net lipid weight to net *P. malhamensis* powder weight was the total lipid content.

The fucoxanthin content of *P. malhamensis* was extracted using ethanol and analyzed with high-performance liquid chromatography (HPLC) according to the method of [33]. In brief, a freeze-dried *P. malhamensis* sample (10 mg) was dissolved with 2 mL absolute ethanol in a brown bottle. The sample solution was then incubated at 45 °C for 4 h with a shaking every 30 min. After filtration with a membrane filter (pore size 0.22 μm), the sample was analyzed with a Waters Alliance e2695 HPLC (Waters Corporation, Milford, MA, USA).

The content of hydrolyzed amino acid was determined with a method modified from [34]. A freeze-dried *P. malhamensis* sample (30–50 mg) was weighed and transferred into an acid hydrolysis tube. Then, 10 mL hydrochloric acid (6 mol L^−1^) was added to hydrolyze the sample, which was held at 110 °C for 24 h under the protection of nitrogen gas. The hydrolyzed sample was then evaporated using nitrogen gas. The dried hydrolysate was resuspended with double-distilled water. The resuspended sample was filtered with a membrane filter (pore size 0.22 μm) and measured using an amino acid analyzer (A300, MembraPure, Berlin, Germany).

The fatty acid composition of *P. malhamensis* was determined using the method of in situ transesterification as previously described [35] with a slight modification. A freeze-dried *P. malhamensis* sample (5 mg) was solubilized with 200 μL of chloroform:methyl alcohol (2:1, *v/v*) and transesterified with 300 μL HCl:MeOH (5%, *v/v*). Then, 25 μL of tridecanoic acid (0.2 mg mL^−1^) was added as the standard substance. The reaction was carried out in a water bath at 85 °C for 1 h. The reactant was then cooled at room temperature for 30 min. The fatty acid methyl esters (FAMEs) thus formed were extracted using 1 mL n-hexane at room temperature for 1–4 h until two phases had been formed. The upper phase (200 μL) containing FAMEs was transferred into a new vial. Pentadecane (5 μL, 0.2 mg mL^−1^) was added to the vial as the internal standard. The analysis was carried out using a gas chromatograph with a flame ionization detector (7890B-5977A, Agilent, Santa Clara, CA, USA) and an HP-88 capillary column. The initial column temperature was set at 50 °C for 2 min and gradually increased to 175 °C for 5 min at rate of 25 °C min^−1^, followed by a rise to 210 °C for 2 min at rate of 7 °C min^−1^, and finally increased to 230 °C for 1 min at rate of 2 °C min^−1^. The injector temperature was set at 250 °C.

### 2.6. Grazing Abilities of Paramecium caudatum on P. malhamensis Cells with Different Trophic Modes

*Poterioochromonas malhamensis*, cultivated with the methods described in Section 2.3 to promote three different nutritional modes (i.e., autotrophy, chemoheterotrophy, and phagotrophy), was used as the prey of *Paramecium caudatum*. The *P. caudatum* originated from the China Zebrafish Resource Center (CZRC, http://en.zfish.cn/ (accessed on 18 April 2022)) and was cultivated using boiled rice straw (10–30 g rice straw per 1 L distilled water). The feeding experiment was performed in 50 mL flasks, with triplicate flasks for each nutritional mode of *P. malhamensis*. An aliquot of supernatant (20 mL) of boiled rice straw containing *P. caudatum* (110 cells mL^−1^) was added to each flask. *Poterioochromonas malhamensis* cells for each nutritional mode were harvested at 1500× *g* for 5 min and then added to these flasks with a final cell concentration of 10^6^ cells mL^−1^. After co-culture for 24 h, the remaining *P. malhamensis* cells were counted using a hemocytometer under light microscopy. The clearance rate (η, %) of prey *P. malhamensis*, calculated according to the method of [31], was used to evaluate the grazing ability of *P. caudatum* on *P. malhamensis*. The clearance rate was calculated using the equation: η = (1 − N_t_/N_0_) × 100%, where N_t_ and N_0_ are the cell concentrations of *P. malhamensis* after and before the grazing experiment, respectively. The experiment was carried out in a culture room with a temperature of 22–25 °C and continuous illumination at a light intensity of 20–30 μmol photons m^−2^ s^−1^.

### 2.7. Statistical Analyses

Differences in biochemical composition between the three nutritional modes of *P. malhamensis* were tested with one-way analysis of variance (ANOVA) followed by the Tukey honestly significant difference test. The correlation between the grazing ability of *Paramecium caudatum* on *P. malhamensis* cells and the physiological/biochemical parameters of *P. malhamensis* was analyzed using Pearson’s correlation coefficient for normally distributed data and Spearman’s rank correlation coefficient for non-normally distributed data. The normality of data was analyzed by the Shapiro–Wilk test. The physiological/biochemical parameters analyzed included cell size, total protein, total lipid, total sugar, total amino acid, total absolute/relative essential amino acids, total absolute/relative non-essential amino acids, total fatty acid, total absolute/relative saturated fatty acids, total absolute/relative monounsaturated fatty acid, and total absolute/relative polyunsaturated fatty acid of *P. malhamensis* cells. Among these parameters, cell size, absolute/relative monounsaturated fatty acid, and relative polyunsaturated fatty acid were non-normally distributed data. Data were analyzed using the SPSS 18.0 software platform.

## 3. Results

### 3.1. Purification of P. malhamensis Culture

**Figure 1:**
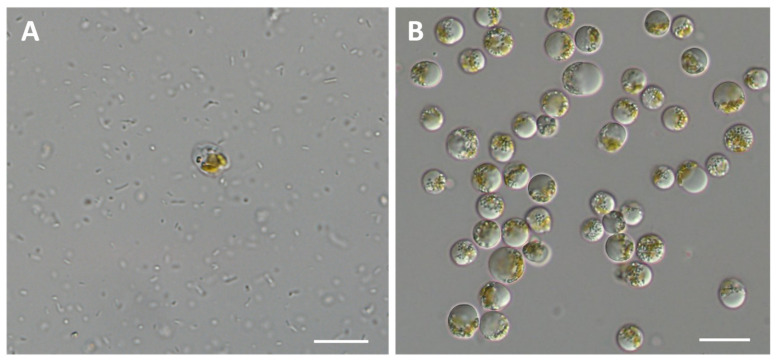
Microscopic observations of non-axenic (**A**) and axenic (**B**) *P. malhamensis* cultivated with enriched medium. Scale bars = 20 μm.

When untreated non-axenic *P. malhamensis* was cultivated using the enriched medium, the contaminant bacteria multiplied greatly and resulted in the cessation of *P. malhamensis* growth (Figure 1A). After repeated treatments using multifarious antibiotics, the *P. malhamensis* culture grew well in the enriched medium, and no bacteria were observed under the microscope (Figure 1B). This indicated that an axenic *P. malhamensis* culture had been obtained.

### 3.2. Growth and Cell Morphology of P. malhamensis with Different Nutritional Modes

**Figure 2:**
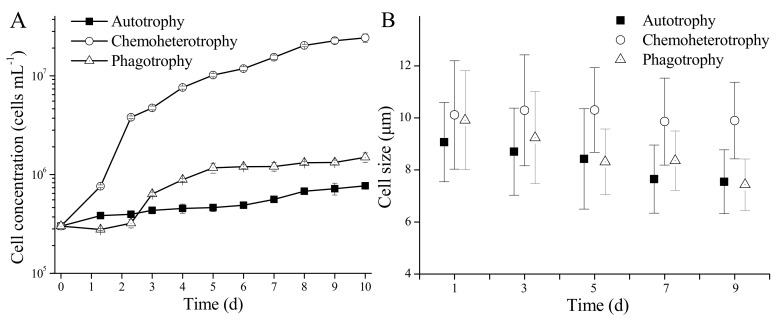
Growth (**A**) and cell size changes (**B**) of *P. malhamensis* with different nutritional modes. Error bars indicate the standard deviation. *n* = 3.

The growth of axenic *P. malhamensis* differed depending on its nutritional mode (Figure 2A). With autotrophy, the cell concentration of *P. malhamensis* increased to 7 × 10^5^ cells mL^−1^ from the initial cell concentration of 3 × 10^5^ cells mL^−1^ after a 10-day cultivation period. However, the cell concentration of *P. malhamensis* reached up to 1 × 10^6^ cells mL^−1^ when prey *Chlorella* was present (i.e., phagotrophy). Furthermore, the cell concentration of *P. malhamensis* under chemoheterotrophic conditions increased to 1 × 10^7^ cells mL^−1^ after cultivation for 4–5 days. This showed that the growth rate of *P. malhamensis* was better with chemoheterotrophy than with autotrophy or phagotrophy. Changes in *P. malhamensis* cell size varied between the different nutritional modes. The cell size of chemoheterotrophic *P. malhamensis* basically remained around the initial value of 10 μm during the whole cultivation period, while the cell sizes of autotrophic and phagotrophic *P. malhamensis* both decreased gradually to 7.5 μm after cultivation for 9 days (Figure 2B).

**Figure 3:**
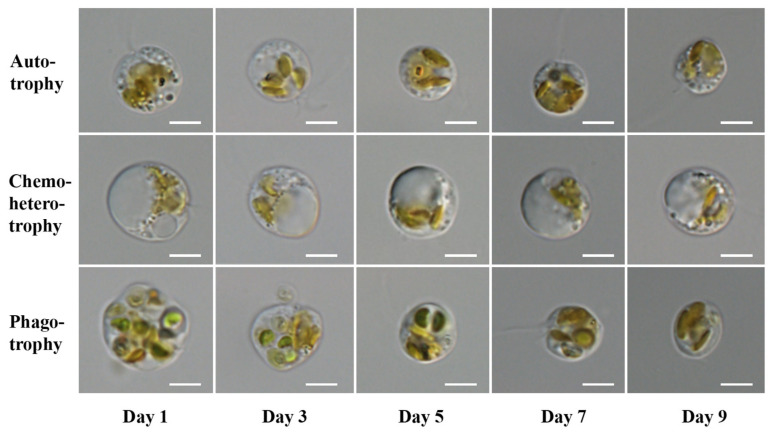
Cell morphologies of *P. malhamensis* with different nutritional modes at different times during cultivation. Scale bar = 5 μm.

However, compared to autotrophic *P. malhamensis*, the cell size of phagotrophic *P. malhamensis* decreased slowly in the first 3 days because of its ability to graze on prey *Chlorella* cells (Figure 3). The cell morphology of *P. malhamensis* also varied greatly between different nutritional modes (Figure 3). The chloroplasts of autotrophic and phagotrophic *P. malhamensis* in the later period of culture (after 7 days) were intact and distinct, while the chloroplasts of chemoheterotrophic *P. malhamensis* over the whole cultivation period were rather amorphous. However, one big vacuole, which occupied more than two-thirds of the total cell volume, was always observed in chemoheterotrophic *P. malhamensis* cells.

### 3.3. Biochemical Composition of P. malhamensis with Different Nutritional Modes

The total lipid content of *P. malhamensis* was similar for all three nutritional modes, all being ca. 28% (Table 1). However, the total protein, total sugar, and fucoxanthin contents of *P. malhamensis* varied greatly between different nutritional modes. The total protein content of autotrophic *P. malhamensis* was higher than that of chemoheterotrophic *P. malhamensis* and phagotrophic *P. malhamensis* (ANOVA, *p* < 0.01). The same trend occurred for fucoxanthin content, with autotrophic *P. malhamensis* exhibiting higher percentages of fucoxanthin than chemoheterotrophic *P. malhamensis* and phagotrophic *P. malhamensis* (ANOVA, *p* < 0.01). However, the total sugar content of *P. malhamensis* was lower with autotrophy than with chemoheterotrophy and phagotrophy (ANOVA, *p* < 0.01).

With respect to the absolute amino acid content (Table 2), the total amino acid content of autotrophic *P. malhamensis* was higher than that of chemoheterotrophic *P. malhamensis* and phagotrophic *P. malhamensis* (ANOVA, *p* < 0.05), which corresponded to the higher total protein content. The contents of total essential amino acids (EAAs) and total non-essential amino acids (NEAAs) in autotrophic *P. malhamensis* cells were also both higher than those in chemoheterotrophic *P. malhamensis* cells and phagotrophic *P. malhamensis* cells. Interestingly, the relative amino acid content for total EAAs and total NEAAs was the same for all three trophic modes, all being around 50%. Furthermore, the relative contents of most amino acids were similar for the three modes, except for arginine in phagotrophic *P. malhamensis*, where the relative content was nearly two times higher than that in chemoheterotrophic *P. malhamensis* and autotrophic *P. malhamensis* (ANOVA, *p* < 0.01). With all three nutritional modes, the amino acid with the richest content in *P. malhamensis* cells was always glutamic acid.

The fatty acid contents of different nutritional *P. malhamensis* cells were different from the results of total lipid content (Table 1 and Table 3). The absolute content of total fatty acid in autotrophic *P. malhamensis* was much lower than that in chemoheterotrophic *P. malhamensis* and phagotrophic *P. malhamensis* (ANOVA, *p* < 0.01). The difference in total fatty acid was mainly caused by the diverse contents of saturated fatty acids (SFAs), with the absolute content of SFAs in autotrophic *P. malhamensis* being much lower than that in chemoheterotrophic *P. malhamensis* and phagotrophic *P. malhamensis* (ANOVA, *p* < 0.01) (Table 3). To be more specific, chemoheterotrophic *P. malhamensis* was rich in the SFAs C14:0 (6.29%) and C16:0 (4.51%), while phagotrophic *P. malhamensis* mainly contained the SFA C16:0 with a content of 5.17%. The content of monounsaturated fatty acids (MUFAs) in autotrophic *P. malhamensis* (0.48%) was also lower than that in chemoheterotrophic *P. malhamensis* and phagotrophic *P. malhamensis* (both 2.75%). The absolute contents of total polyunsaturated fatty acids (PUFAs) in different trophic *P. malhamensis* cells were all around 10%, while the individual PUFA content in different trophic *P. malhamensis* cells varied greatly. Autotrophic *P. malhamensis* had the lowest absolute content of the PUFA C18:2. The absolute content of the PUFA C18:3 in chemoheterotrophic *P. malhamensis* cells was much lower than that in autotrophic *P. malhamensis* cells and phagotrophic *P. malhamensis* cells (ANOVA, *p* < 0.01). Phagotrophic *P. malhamensis* cells had a lower absolute content of the PUFA C22:5, compared to chemoheterotrophic *P. malhamensis* cells and autotrophic *P. malhamensis* cells. However, with respect to the relative fatty acid content, the relative content of PUFAs in autotrophic *P. malhamensis* cells was much higher than that in chemoheterotrophic *P. malhamensis* and phagotrophic *P. malhamensis* (ANOVA, *p* < 0.01).

### 3.4. Grazing Abilities of Paramecium caudatum on P. malhamensis with Different Trophic Modes

**Figure 4:**
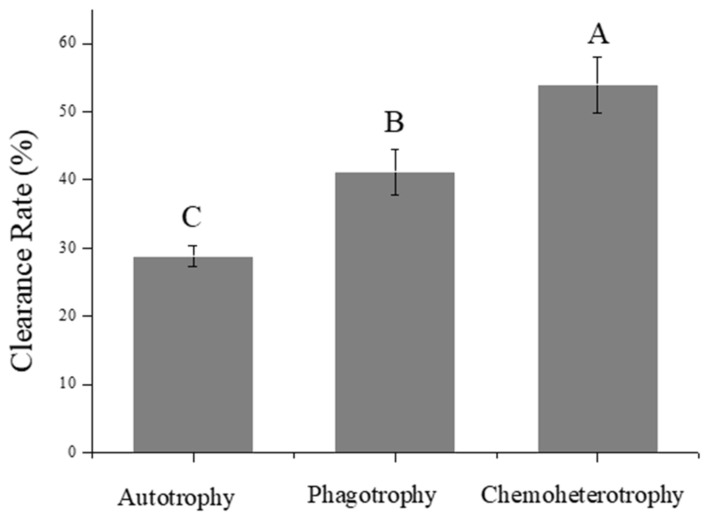
Clearance rate of *Paramecium caudatum* on different trophic *P. malhamensis* cells. Significant differences are indicated by different uppercase letters (one-way ANOVA, *p* < 0.01). Error bars indicate the standard deviation. *n* = 3.

The clearance rate of *P. caudatum* on chemoheterotrophic *P. malhamensis* cells (53.88%) was significantly higher than that on phagotrophic *P. malhamensis* cells (41.17%) and autotrophic *P. malhamensis* (28.86%) (ANOVA, *p* < 0.01) (Figure 4). This showed that the grazing ability of *P. caudatum* on *P. malhamensis* cells differs depending on the nutritional mode of the *P. malhamensis*.

**Figure 5:**
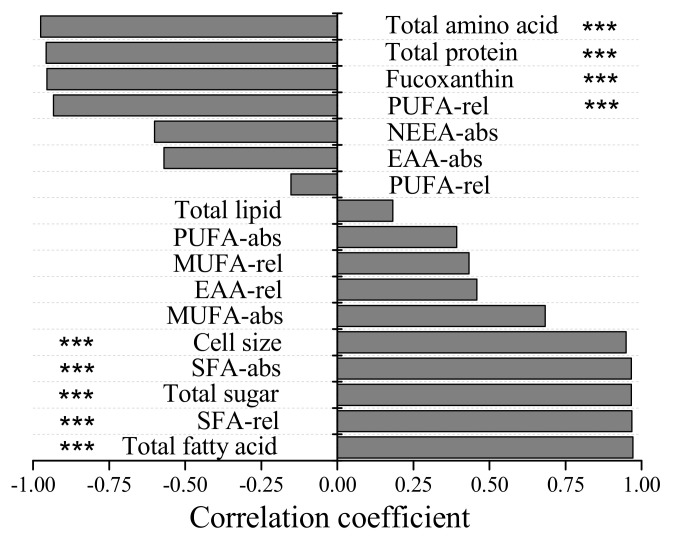
Correlation analysis between clearance rate of *Paramecium caudatum* on *P. malhamensis* cells with different nutritional modes and various physiological and biochemical parameters of *P. malhamensis*. Significant differences are indicated by three asterisks (*p* < 0.001).

Correlation analysis showed that the grazing ability of *P. caudatum* on *P. malhamensis* cells had significant negative correlations (*r* < −0.95, *p* < 0.001) with the total amino acid, total protein content, total relative PUFA content, and fucoxanthin content of *P. malhamensis* cells (Figure 5). On the other hand, the grazing ability of *P. caudatum* on *P. malhamensis* cells had significant positive correlations (*r* > 0.95, *p* < 0.001) with the total fatty acid content, total relative SFA content, total sugar content, total absolute SFA content, and cell size of *P. malhamensis* cells.

## 4. Discussion

### 4.1. Purification and Cultivation of P. malhamensis

Obtaining an axenic culture of *P. malhamensis* was a critical step in studying the nutritional modes of this mixotrophic flagellate. However, as most mixotrophic flagellates lack a cell wall and cannot grow well on solid media, axenic seed cultures of mixotrophic flagellates are difficult to obtain. Therefore, the mixotrophic flagellate cultures involved in some studies are actually not axenic [36]. In our study, an axenic *P. malhamensis* seed culture was obtained through repeated treatments using multifarious antibiotics. The axenic *P. malhamensis* culture allowed a more precise study of the physiological and biochemical characteristics of *P. malhamensis*. Looking beyond the present study, having an axenic seed culture of *P. malhamensis* also makes it possible to exploit the flagellate’s commercial application value. *Poterioochromonas malhamensis* could be used to produce antimicrobial malhamensilipin A [37], active polysaccharides β-1,3-glucan [38], or even to control *Microcystis* blooms [39]. However, its application values have been restricted greatly by the undeveloped mass cultivation methods commonly used for *P. malhamensis*. In this study, the maximum cell concentration of *P. malhamensis* was in the order of magnitude of 10^7^ cells mL^−1^, but we have established a chemoheterotrophic fermentation technology for cultivation of *P. malhamensis* using the axenic seed culture, which yields a maximum cell concentration and dry weight of *P. malhamensis* under optimal fermentation conditions of 3 × 10^8^ cells mL^−1^ [40] and 32.8 g L^−1^ [38], respectively.

Our study showed that *P. malhamensis* exhibited an extremely low growth rate when relying upon autotrophy (Figure 2A), which is a similar finding to that of a previous study [28], which indicates that the photosynthetic ability of *P. malhamensis* is low. The low photosynthetic ability of *P. malhamensis* is speculated to result from the lack of a CO_2_ concentrating mechanism as in other species of chrysophyte [41]. The nutritional mode of *P. malhamensis* was once defined as being predominately phagotrophic mixotrophy, which means that it can achieve its maximum specific growth rate (*μ*_max_) only when particulate prey is provided [4]. However, a higher *μ*_max_ can be observed when *P. malhamensis* is cultivated with dissolved organic matter [42], and this is consistent with our results. It is notable that the total organic carbon concentrations under phagotrophic conditions and chemoheterotrophic conditions in different studies were different. Therefore, it is more appropriate to describe the nutrition mode of *P. malhamensis* as being ‘predominately heterotrophic mixotrophy’.

### 4.2. Effects of Nutritional Mode on the Morphological and Biochemical Characteristics of P. malhamensis

Over the whole cultivation period, the cell size of *P. malhamensis* under chemoheterotrophic conditions was always larger than that under autotrophic conditions, this resulting from the formation of a bulky chrysolaminarin vesicle (Figure 3) containing a large amount of β-1,3-glucan as the storage substance [38]. The same phenomenon has also been observed in another chrysophyte, *Ochromonas* [43]. The mixotrophic microalga *Chlorella sorokiniana* has also been found to increase its cell volume when using heterotrophy by accumulating an excess of storage starch [44]. On the other hand, the protein and total amino acid contents of autotrophic *P. malhamensis* were both significantly higher than those of chemoheterotrophic *P. malhamensis* (ANOVA, *p* < 0.05). This indicates that the storage substance chrysolaminarin might be utilized to synthesize other functional biomolecules (e.g., protein and polypeptides) when *P. malhamensis* transforms from chemoheterotrophy to autotrophy.

With respect to pigment content, it has been widely proved that chlorophyll concentrations of the mixotrophic flagellates *Poterioochromonas* or *Ochromonas* decline after the addition of organics to the culture medium [28,42,45]. Correspondingly, in the present study, *P. malhamensis* cells under chemoheterotrophic conditions exhibited a pale chloroplast color and a low content of fucoxanthin (Figure 3 and Table 1), which indicated that the photosynthetic system of heterotrophic *P. malhamensis* cells might be closed when exogenous organic matter is available.

To date, the biochemical composition of *P. malhamensis*, especially in terms of amino acids and fatty acids, has rarely been studied. Essential amino acids are those that animals or humans cannot synthesize de novo and so can only obtain from a food source. However, most plant cells can synthesize all kinds of amino acids de novo [46]. In the present study (Table 2), all the amino acids were present at higher ratios in autotrophic *P. malhamensis* than in chemoheterotrophic *P. malhamensis*, which showed that autotrophic *P. malhamensis* can synthesize all amino acids de novo. Therefore, the mixotrophic *P. malhamensis* cells are more like plant cells from the perspective of biochemical characteristics. With respect to fatty acids, PUFAs were considered in a previous study [36] to be the most reliable biochemical variable at indicating the nutritional mode of *Ochromonas* sp., since the total absolute PUFA contents of *Ochromonas* sp. varied greatly between different nutritional modes. In contrast, the total absolute PUFA contents in *P. malhamensis* cells with different trophic modes were the same (Table 3), which indicated that *Poterioochromonas* and *Ochromonas* might have different PUFA metabolism pathways. In contrast, the total absolute SAF content of autotrophic *P. malhamensis* was lower than that of heterotrophic *P. malhamensis*, which was consistent with the variations observed in *Ochromonas* sp. [36]. Therefore, it is likely that the SAFs are another form of storage in *P. malhamensis* and could be used as one of the biochemical variables reflecting the nutritional mode of *P. malhamensis*.

### 4.3. Effects of Variation in the Biochemical Composition of P. malhamensis, Resulting from Different Nutritional Modes, on Its Predators’ Feeding Behavior and the Implications for Aquatic Food Webs

Recently, the important roles of mixotrophic flagellates in connecting the classical food chain and microbial loop have attracted more and more attention from ecologists [47,48]. The present study has shown that the transformation of nutritional modes can alter the biochemical composition of the mixotrophic flagellate *P. malhamensis* and, as a result, affect the grazing ability of its predator *Paramecium caudatum*. Moreover, a recent study has shown that the grazing ability of *P. malhamensis* on its prey *Microcystis aeruginosa* also varies depending on the nutritional mode of the *P. malhamensis* [40]. These studies imply that changes in environmental nutrient resources would greatly affect the flow directions within food chains centered on mixotrophic flagellates. However, it is difficult to be sure about the precise causal relationships between apparent grazing abilities of predators and nutritional modes of prey. In the present study, Pearson’s correlation analysis showed that the grazing ability of *P. caudatum* on *P. malhamensis* cells is positively correlated with the content of fatty acids (especially the SFAs) in *P. malhamensis*. As certain fatty acids (e.g., SFA C18:0) have generally been considered as indispensable nutrients in artificially synthesized media for *Paramecium* [49], we have been convinced to some extent that the stronger grazing ability of the ciliate *P. caudatum* on chemoheterotrophic *P. malhamensis* cells may result from the higher content of SFAs in chemoheterotrophic *P. malhamensis* cells. In addition, our analytical results also showed that the ciliate *P. caudatum* was seemingly better at grazing on chemoheterotrophic *P. malhamensis* with a high content of chrysolaminarin (i.e., β-1, 3-glucan). To date, the ability of *P. caudatum* to utilize chrysolaminarin remains uncertain, while a previous study [50] revealed that the addition of the storage substance glycogen could also improve the growth of *Paramecium*. However, it is notable that most biochemical variables are correlated with each other. For instance, increasing contents of total sugar and lipid are generally accompanied by a decreased content of protein. Therefore, the correlations between grazing ability and any one biochemical variable will be affected by the internal interactions between different biochemical variables. So, although the correlation results in the present study showed that the grazing ability of *P. caudatum* on *P. malhamensis* had significant negative correlations with the protein (or amino acid) content and relative PUFA content of *P. malhamensis* cells, this does not necessarily mean that there is a direct causal link between grazing ability and protein or PUFA content. Indeed, amino acids and PUFA C18:2 have always been considered as important nutrients supporting the growth of *Paramecium* [49]. Therefore, it is still difficult for us to judge which biochemical variable of *P. malhamensis* is the main or the real factor affecting *P. caudatum* grazing on *P. malhamensis* with different nutritional modes.

The present study has provided a case report illustrating that changes in environmental resources (presented by different nutritional modes) can alter the biochemical composition of a mixotrophic flagellate and, as a consequence, affect the grazing ability of its predator. However, in natural ecosystems, the mixotrophic flagellate *P. malhamensis* could also be grazed by many other zooplankton species, such as *Daphnia magna* and *Brachionus angularis* [16,51], and the altered biochemical composition of *P. malhamensis* may bring about more complicated influences on diverse higher trophic predators that have different nutritional requirements. Conceivably, some consumers (e.g., *P. caudatum* in this study) will prefer to graze on chemoheterotrophic *P. malhamensis* cells rich in sugar and fatty acid, while other consumers may show no interest in feeding on chemoheterotrophic *P. malhamensis* cells because of their low amino acid content. This speculation is supported by a previous study, which revealed that the feeding preferences of various rotifers (including *Brachionus sericus*, *Cephalodella* sp., and *Elosa worallii*) for autotrophic, heterotrophic, and mixotrophic *Chlamydomonas acidophila* cells differed between the rotifer species [11]. Given these complex influences, we therefore suppose that the direction of carbon transfer and energy flow in aquatic food webs centered on mixotrophic flagellates may be altered as a result of changes to the nutritional mode used by these flagellates and the concomitant changes in their biochemical composition.

## 5. Conclusions

The physiological and biochemical composition of axenic *P. malhamensis* cells, which was obtained by use of multifarious antibiotics, was closely related to the nutritional mode being used by the *P. malhamensis* cells. Under chemoheterotrophic conditions, *P. malhamensis* cells exhibited the highest growth rate and largest cell size and simultaneously had the highest content of total sugar as well as total absolute fatty acid. Autotrophic *P. malhamensis* cells exhibited the lowest growth rate and smallest cell size but had the highest content of protein, total absolute amino acid, and fucoxanthin. The contents of most biochemical components in phagotrophic *P. malhamensis* cells fell between those in autotrophic and chemoheterotrophic *P. malhamensis* cells. A feeding experiment showed that *P. caudatum* was better at grazing on chemoheterotrophic *P. malhamensis* cells, followed by phagotrophic and then autotrophic *P. malhamensis*. These results indicate that changes in nutritional mode can alter the biochemical composition of the mixotrophic flagellate *P. malhamensis*, and this, in turn, can affect the grazing ability of its predator *Paramecium caudatum*. Further studies should focus on collecting more field evidence to verify the effect of environmental changes on the direction of carbon transfer and energy flow in aquatic food webs centered on mixotrophic flagellates.

## Figures and Tables

**Table 1:** Biochemical composition of *P. malhamensis* cultivated with different nutritional modes (*n* = 3).

	Total Lipid (%)	Total Protein (%)	Total Sugar (%)	Fucoxanthin (%)
Autotrophy	28.2 ± 0.8	42.1 ± 1.0	11.3 ± 0.5	0.34 ± 0.03
Chemoheterotrophy	28.4 ± 0.6	23.0 ± 1.6	42.8 ± 3.4	0.12 ± 0.00
Phagotrophy	27.4 ± 0.3	30.1 ± 0.8	24.7 ± 1.1	0.20 ± 0.01

**Table 2:** Amino acid composition of *P. malhamensis* with different nutritional modes (*n* = 3).

Amino Acid		% of Dry Weight (Absolute)			% of Total Amino Acid (Relative)		
		Auto	Chemo	Phago	Auto	Chemo	Phago
Essential amino acid (EAA)	Thr	1.80 ± 0.00	1.11 ± 0.05	1.43 ± 0.07	5.31 ± 0.01	5.15 ± 0.22	4.95 ± 0.24
	Val	2.01 ± 0.06	1.22 ± 0.03	1.57 ± 0.05	5.93 ± 0.19	5.67 ± 0.14	5.42 ± 0.19
	Met	0.39 ± 0.02	0.12 ± 0.02	0.35 ± 0.05	1.15 ± 0.66	0.57 ± 0.07	1.21 ± 0.16
	Ile	1.72 ± 0.06	1.14 ± 0.08	1.11 ± 0.11	5.06 ± 0.16	5.31 ± 0.37	3.84 ± 0.37
	Leu	3.02 ± 0.04	1.97 ± 0.08	2.23 ± 0.15	8.88 ± 0.11	9.18 ± 036	7.69 ± 0.51
	Phe	2.19 ± 0.04	1.41 ± 0.03	1.59 ± 0.08	6.45 ± 0.13	6.55 ± 0.14	5.48 ± 0.28
	His	0.96 ± 0.04	0.71 ± 0.02	0.66 ± 0.03	2.82 ± 0.11	3.29 ± 0.10	2.28 ± 0.10
	Lys	2.70 ± 0.04	1.78 ± 0.06	1.73 ± 0.09	7.90 ± 0.11	8.30 ± 0.27	5.98 ± 0.32
	Arg	2.15 ± 0.08	1.31 ± 0.06	3.35 ± 0.13	6.30 ± 0.22	6.10 ± 0.29	11.57 ± 0.46
	Total	16.94 ± 0.12	10.78 ± 0.31	14.04 ± 0.74	49.80 ± 0.34	50.11 ± 1.42	48.41 ± 2.56
Non-essential amino acid (NEAA)	Asp	4.21 ± 0.10	2.53 ± 0.05	3.27 ± 0.19	12.38 ± 0.29	11.76 ± 0.24	11.27 ± 0.65
	Ser	1.77 ± 0.05	1.12 ± 0.05	1.31 ± 0.07	5.21 ± 0.15	5.23 ± 0.23	4.52 ± 0.24
	Glu	4.23 ± 0.09	2.87 ± 0.12	4.26 ± 0.18	12.42 ± 0.27	13.33 ± 0.56	14.70 ± 0.61
	Gly	1.77 ± 0.06	1.14 ± 0.05	1.59 ± 0.07	5.20 ± 0.19	5.32 ± 0.22	5.49 ± 0.23
	Ala	2.13 ± 0.04	1.26 ± 0.04	2.06 ± 0.12	6.24 ± 0.12	5.88 ± 0.20	7.11 ± 0.40
	Cys	0.14 ± 0.01	0.06 ± 0.03	0.18 ± 0.05	0.27 ± 0.24	0.30 ± 0.12	0.62 ± 0.17
	Tyr	1.27 ± 0.04	0.76 ± 0.04	1.04 ± 0.03	3.72 ± 0.12	3.55 ± 0.18	3.60 ± 0.11
	Pro	1.62 ± 0.12	0.97 ± 0.05	1.24 ± 0.05	4.76 ± 0.34	4.51 ± 0.22	4.28 ± 0.19
	Total	17.14 ± 0.31	10.73 ± 0.34	14.96 ± 0.72	50.20 ± 0.91	49.89 ± 1.58	51.59 ± 2.48
Total		34.08 ± 0.31	21.51 ± 0.63	29.00 ± 1.44	100	100	100

Note: Thr, Threonine; Val, Valine; Met, Methionine; Ile, Isoleucine; Leu, Leucine; Phe, Phenylalanine; His, Histidine; Lys, Lysine; Arg, Arginine; Asp, Aspartic acid; Ser, Serine; Glu, Glutamic acid; Gly, Glycine; Ala, Alanine; Cys, Cystine; Tyr, Tyrosine; Pro, Proline. Auto, Chemo, and Phago represent autotrophy, chemoheterotrophy, and phagotrophy, respectively.

**Table 3:** Fatty acid composition of *P. malhamensis* with different nutritional modes (*n* = 3).

Fatty Acid	%, of Dry Weight (Absolute)			%, of Total Fatty Acid (Relative)		
	Auto	Chemo	Phago	Auto	Chemo	Phago
C14:0	1.66 ± 0.04	6.29 ± 0.64	1.18 ± 0.10	12.20 ± 0.16	24.38 ± 0.06	5.74 ± 0.34
C15:0	0.02 ± 0.00	0.01 ± 0.00	0.03 ± 0.00	0.14 ± 0.01	0.02 ± 0.01	0.14 ± 0.00
C16:0	1.36 ± 0.03	4.51 ± 0.45	5.17 ± 0.13	10.06 ± 0.13	17.49 ± 0.01	25.07 ± 0.69
C17:0	0.03 ± 0.00	0.04 ± 0.00	0.08 ± 0.00	0.21 ± 0.01	0.16 ± 0.01	0.39 ± 0.01
C18:0	0.14 ± 0.00	2.01 ± 0.20	0.86 ± 0.02	1.03 ± 0.02	7.80 ± 0.01	4.16 ± 0.09
C22:0	0.08 ± 0.00	0.10 ± 0.01	0.05 ± 0.01	0.56 ± 0.03	0.39 ± 0.01	0.24 ± 0.02
∑SFA	3.29 ± 0.08	12.96 ± 1.31	7.37 ± 0.25	24.22 ± 0.35	50.24 ± 0.11	35.74 ± 1.15
C16:1	0.05 ± 0.00	0.49 ± 0.04	0.16 ± 0.00	0.40 ± 0.02	1.89 ± 0.02	0.78 ± 0.04
C17:1	0.01 ± 0.01	0.01 ± 0.01	0.08 ± 0.00	0.078 ± 0.09	0.04 ± 0.02	0.38 ± 0.01
C18:1	0.19 ± 0.00	1.47 ± 0.13	2.30 ± 0.07	1.41 ± 0.05	5.71 ± 0.08	11.15 ± 0.41
C22:1	0.23 ± 0.01	0.78 ± 0.09	0.21 ± 0.03	1.68 ± 0.07	3.02 ± 0.05	1.03 ± 0.12
∑MUFA	0.48 ± 0.04	2.75 ± 0.27	2.75 ± 0.11	3.56 ± 0.24	10.66 ± 0.18	13.35 ± 0.57
C18:2	2.44 ± 0.07	5.56 ± 0.55	5.38 ± 0.14	18.01 ± 0.13	21.57 ± 0.01	26.11 ± 0.14
C18:3	3.39 ± 0.08	1.02 ± 0.10	3.64 ± 0.19	24.96 ± 0.08	3.94 ± 0.01	17.67 ± 0.50
C18:4	0.14 ± 0.00	0.06 ± 0.01	0.03 ± 0.01	1.02 ± 0.01	0.22 ± 0.01	0.16 ± 0.02
C20:5	0.45 ± 0.01	0.25 ± 0.02	0.23 ± 0.02	3.33 ± 0.05	0.98 ± 0.01	1.09 ± 0.06
C22:5	2.99 ± 0.07	2.97 ± 0.30	0.99 ± 0.08	22.01 ± 0.29	11.50 ± 0.03	4.79 ± 0.29
C22:6	0.39 ± 0.01	0.23 ± 0.01	0.22 ± 0.00	2.87 ± 0.06	0.89 ± 0.03	1.07 ± 0.01
∑PUFA	9.80 ± 0.25	10.09 ± 1.00	10.49 ± 0.43	72.21 ± 0.61	39.10 ± 0.10	50.89 ± 1.03
Total	13.57 ± 0.32	25.80 ± 2.57	20.61 ± 0.56	100	100	100

Note: SFA, saturated fatty acid; MUFA, monounsaturated fatty acid; PUFA, polyunsaturated fatty acid. Auto, Chemo, and Phago represent autotrophy, chemoheterotrophy, and phagotrophy, respectively.

## Data Availability

Not applicable.
